# Divinity vs. Physic

**Published:** 1854-05

**Authors:** 


*Divinity* vs. *Physic.*— Our thoughts have been recently directed to the relations of the two learned professions, whose names stand above, by reading in a religious weekly newspaper, edited by an esteemed clerical friend, a "first-rate notice" of a water-cure establishment, a mongrel house of healing, where the opposite doctrines of homœopathy, and hydropathy, are made to harmonize under the direction of a German quack, who claims to have graduated at sundry schools in the Fatherland, but who hails last as a somewhat distinguished graduate from a "State Institution" on this side of the Atlantic, a "Government School" located at the easterly end of Cayuga bridge, where dietetics and exercise are particularly enforced, its inmates being confined to mush and molasses for the one, and to stone-cutting and shoe-making for the other.

This attending physician, a man known by the editor, and a majority of his readers, to be a jail-bird, a man of low morals in his daily life, and a criminal upon occasion, abhorred by the virtuous, convicted by jurymen, sentenced by judges, cropped, shaved, and dressed in striped clothes by turnkeys, is now, upon his release from durance vile, considered a fit subject for a laudatory notice in a denominational paper of large circulation. We are aware that among that class of sisters who resort to these watercures, or dally with the sweet morsels of homœopathy, a taint of rascality in their physician is rather relished than otherwise. All the long category of "reformers," whether in medicine, morals, or politics, manifest a similar predilection; a sneaking tenderness for great sinners; a warm sympathy for the *unfortunate* inmates of jails and prisons; while ragged virtue walks the streets, uncared-for and unnoticed. But we had not supposed that our good friend whose really excellent paper contains this puff, was one to fall in with such a weakness. It is probable that it was a mere piece of good-nature on his part, a sort of clincher to a long and profitable advertisement that decked another [redacted]

We will not, therefore, look at the matter in a personal light, nor lay it at the door of one whom we know to be a truly pious and worthy man. We prefer rather to run over, briefly, the relative situations of divinity and medicine, to inquire into their proper spheres of action, and state the causes of the decay of that comity which once existed between them. It is known that a breach has opened, which has grown wider in the progress of events in an age of moral changes.

Were we to confine these remarks only to an ideal of what should be the intercourse of the divine and the physician, we should simply refer our readers to that sermon which we noticed and quoted in our issue for March, as an example of what is needed by both professions. But it happens that the Rev. Dr. Lord, though a strong, sensible, and right reasoning man in this regard, is not a true exponent of the feelings of his brethren.

The efficiency and success of the ministry of Christ rests in no small measure upon the support which it derives from the other learned professions. Its claims are, in these latter days, freely discussed in the light of scientific truth. The sure deductions of the natural sciences come up occasionally, in apparent contradiction to the doctrines of the pulpit. It happens that the medical profession is now, and, in all probability ever will be the reservoir in which the facts of science are aggregated, and its members are, therefore, their principal exponents. To it and its teachings must divinity look for the necessary confirmation of the truths of revelation.

In the present state of religious belief, when the doctrines of the French encyclopedists (so abhorred and shunned a few score years ago) are becoming wide-spread, when infidelity no longer cloaks itself in darkness, or hides among the vicious and unlearned, it is necessary that divinity should call to its aid all of those means which may best stem the tide of unbelief. For deism, and atheism even, are not now confined to a few crack-brained and wicked men. They find a place in the saloons of the rich, in the social circle of the well-informed, in the hearts of the moral and virtuous in life. It is a wide-spread contagion, infecting every part of society, and entering within the pale of the church itself.

We recollect to have read, a few years since, a certain novel called "Yeast," a name which was chosen as typifying the fermenting theories of the present day. Whether intentionally or not, we cannot say, but its author embodied a great truth in the incidental workings of the plot. The religious heresies of the day were so commingled with heresies in medicine, that they appeared as one manifestation. Let us look at this, and see how far it holds true in American life.

We can guess a man's religious creed with tolerable accuracy, if we know his medical tendencies. Search our country over, and it will be found that homœopathy, hydropathy, spiritual rappings, clairvoyance, mesmerism, and all the other forms of quackery, are but the expressions of a spirit of unbelief; the evidence and effect of an infidel feeling. The same peculiar organization of mind which makes the homœopathist, will make the infidel when its attention is directed from physic to religion. The same individuals figure like the supernumeraries on the stage in one eternal round. The Mormon of to-day is the Thomsonian of yesterday, and that precious band of sisters, male and female, who lead in the medical reforms of the day, are also found presiding (if females) in breeches at Women's Rights Conventions, and bolstering up the fooleries of homœopathy and spiritual rappings in the home circle. They are not a class so large in number as one might suppose. They blow their horns all together; they assemble in conventions; they itinerate from city to city, and by thus keeping themselves constantly before the people, they magnify their numbers and importance.

And now let us ask in sad earnest, have the clergy looked upon this matter rightly? That they have afforded material aid and comfort to the enemies of legitimate medicine is but too evident. They are to be found as gratuitous inmates, or (to use a plainer phrase) as charity patients, in every water-cure hotel in the land. Their letters written thence to religious journals are models of the puff indirect, and generally are filled with arguments, derived from some school-boy philosophy, as to the abounding benefits of that everlasting flood, which, only, is more wishy-washy than their laudatory effusions. Returning to their flocks, our gentle shepherds are found gabbling about from house to house, the story of their cure—a cure, in ninety-nine cases in a hundred, the result of unwonted cleanliness, and vigorous exercise, upon an unsexed and effeminated constitution. They are found, too, among the infinitesimals, praising a theory which they cannot understand, and which, if true, strikes at the root of all logic, and all deduction from cause to effect, which does away with one of nature's first and greatest laws, and only harmonizes with that peculiarly clerical dogma, *credo* QUIA *impossibile est.*

But these remarks apply only to that class of fashionable ministers at the altar of God who seem to have lost all sense of clerical dignity, of the true mission of the priest, who, from long and exclusive association with petticoated humanity, have lost all vigor and virility.

There is another class who look upon the church as a means by which to spoil the Gentiles, as a source of fat salaries, and comfortable living. These are the men who follow the humbugs of the day as the wealthy of their parish may dictate, and whose doctrines always coincide with those of him who stands largest on their subscription list. They lend themselves to little meannesses. If they have a homœopathic flock, they are found dabbling in little pills, or if by chance their people are not given to quackery, they have some favorite regular physician, to whose interests they are willing to prostitute their influence as pastors. Very recently one of these has preached a homœopathic sermon, in which he spoke most movingly of the sanctifying and spiritualizing tendencies of the Hahnemannic globule. Sin, he said, was a disease, and he would have the sheep who had gone astray, subjected to a homœopathic course, preparatory to the meat of the Word. It was our peculiar privilege to listen to a prayer, made by another of these at a funeral, wherein he informed the Omniscient that not all the tender watchings of a devoted husband, *nor all the faithful care of a most skillful and experienced physician*, had been able to rescue from the tomb, the loved form of the sister who lay shrouded before them. And this is not the only instance in which we have known the pulpit, and the public services of the House of God to be prostituted to the advertisement and recommendation of a favorite physician.

It is by such means that an alienation has been effected between two professions which should be found the firmest allies. In spite of the charge so freely made use of from the pulpit and in private intercourse, that the medical profession is universally given to skepticism, we venture the assertion that there is as much true piety, and as much God-like charity in it, as in any other body of equal numbers and intelligence. We say more. We believe, so far as human means can effect the foregone purposes of God, that on the medical profession rests, in a great measure, the safety and permanence of the Christian religion. It has already been declared, that the battle of the evidences is yet to be fought in the pages of the medical journals. Are the clergy willing to trust their cause in the hands of men whom they have abused, slighted, and misrepresented? Can they expect a cordial support from the Christian physician even, when his whole life is declared by them a fraud, and a lie?

One of the most decisive evidences of this division is found in the action of the Connecticut State Medical Society, which passed a resolution, refusing to give their services to the clergy and their families, gratuitously, and assigning as a reason for their departure from this time-honored custom, that the clergy were undeserving of their sympathies, inasmuch as not a quack nostrum in the country was destitute of a string of reverend names, attached to certificates of its virtues.

The society might have placed this matter on a higher ground. They might have said that this system of gratuitous service was a derogation from the dignity of both parties; that it placed the physician in the light of one who bids for influence; while to the clergyman it is injurious, as making him a recipient of benefits unpaid for; and as forming a part of those miscalled charities, which are given to the clergy in lieu of their dues, tending thus to diminish their salaries, and leave them in a condition of dependency on the good will of those toward whom they should have no fear.

There is one further consideration which we wish to offer to our clerical friends.

Physicians are not to be dispensed with in the support of religion. Their influence in spiritual matters outweighs that of the clergy. In the medical profession of the United States is concentrated four-fifths of the learning of the country. Aside from their familiarity with the natural sciences, physicians, as a class, are tolerably posted in logic, history, and mental philosophy. Necessarily, so much acquirement confers a corresponding influence. The regular profession is looked to as authority by the courts of law, and by our civic rulers. It is as strong now as ever, as able to wield an immense influence wherever it may choose to exert it.

The tendencies of this great and learned body of men, is toward that true conservatism of which the Christian religion is the highest embodiment. Thither go their sympathies; the support of morals and religion is as natural to them as their breath. In spite of all the distrust and obloquy thrown upon them, they are still found as constant in their faith as though it was appreciated. But the faith which meets no sympathy or credence is apt to be a silent one. Physicians feel that they are peculiarly independent of clerical influence. There is a great world outside of the church, larger than all denominations put together, which does not stop to inquire about creeds, but only asks for skill and honesty. Naturally the profession turn thither, until worn out by want of confidence, and the constant intermeddlings of officious clergymen in their daily avocations, they turn aside to men of sense who trouble themselves with no questions beyond their power of prehension. Such are the causes of this dissension.

We have, finally, a little text from the Latin, which we wish to commend to all who dictate upon matters of medicine withould the necessary preliminary study. *Ne sutor ultra crepidam*—let the shoemaker stick to his last!

A clergyman should start first with this proposition: That the tendencies of truth will lead no man into heresies. Then we may logically conclude, that all those forms of belief which depart from known and acknowledged truth, and, most of all, *which attract to them, as by chemical affinity, the minds of those who are given to running after the strange doctrines against which Paul warned the church*, contain within themselves some element of wrong. If they apply this rule, they will find that phrenology is wrong, because it tends to materialism; that homœopathy is wrong, because it does not acknowledge the law of cause and effect; that all the humbugs of the day are wrong, because their tendency is to some form of unbelief, a tendency evidenced in the motley groups of all shades and forms of heresy which come up to their support.

Said Dr. Lee: "A person who is ultra in one thing, will be ultra in all; a believer in homœopathy will be, most likely, a believer in spirit-rappings, and mesmerism. Six-sevenths of the followers of Emanuel Swedenborg, it is ascertained, are enthusiastic disciples of Hahnemann. A mystic in religion will be a mystic in medicine."

